# Supplementary material for: Meta-Analysis and Gene Set Enrichment Relative to ER Status Reveal Elevated Activity of MYC and E2F in the “Basal” Breast Cancer Subgroup
Source: PLoS One. 2009 Mar 9;4(3):e4710. doi: 10.1371/journal.pone.0004710 (PMC2650420; doi:10.1371/journal.pone.0004710)
Supplement: Dataset S1 — GSEA results from first screen. The file contains a zipped archive of the HTML results of the second GSEA screen. Reports for enriched or depleted datasets may be accessed from the “index.html” link within the my_analysis.GseaPreranked.1219975950625.rpt.zip ZIP archive. Enrichment plots are available for each of the 50 most enriched and depleted gene sets. (13.32 MB ZIP) [file pone.0004710.s001.zip › my_analysis.GseaPreranked.1219975950625.rpt.zip!//VERNELL_PRB_CLSTR1.html]

Details for gene set VERNELL\_PRB\_CLSTR1[GSEA]

|  || Dataset | zSco.ERneg.to.ERpos\_collapsed |
| Phenotype | NoPhenotypeAvailable |
| Upregulated in class | na\_pos |
| GeneSet | VERNELL\_PRB\_CLSTR1 |
| Enrichment Score (ES) | 0.66200864 |
| Normalized Enrichment Score (NES) | 2.45032 |
| Nominal p-value | 0.0 |
| FDR q-value | 0.0 |
| FWER p-Value | 0.0 |
Table: GSEA Results Summary

  

Fig 1: Enrichment plot: VERNELL\_PRB\_CLSTR1      
 Profile of the Running ES Score & Positions of GeneSet Members on the Rank Ordered List

  

| PROBE | GENE SYMBOL | GENE\_TITLE | RANK IN GENE LIST | RANK METRIC SCORE | RUNNING ES | CORE ENRICHMENT || 1 | CCNE1 |  |  | 24 | 6.716 | 0.0406 | Yes |
| 2 | PTX3 |  |  | 49 | 6.328 | 0.0788 | Yes |
| 3 | BLM |  |  | 56 | 6.255 | 0.1179 | Yes |
| 4 | FANCA |  |  | 99 | 5.866 | 0.1518 | Yes |
| 5 | BTG3 |  |  | 102 | 5.850 | 0.1886 | Yes |
| 6 | SKP2 |  |  | 127 | 5.694 | 0.2227 | Yes |
| 7 | RFC3 |  |  | 164 | 5.493 | 0.2547 | Yes |
| 8 | MCM4 |  |  | 180 | 5.436 | 0.2880 | Yes |
| 9 | MELK |  |  | 181 | 5.424 | 0.3222 | Yes |
| 10 | DEK |  |  | 190 | 5.364 | 0.3555 | Yes |
| 11 | RAD51AP1 |  |  | 267 | 5.028 | 0.3816 | Yes |
| 12 | CDC25A |  |  | 281 | 4.993 | 0.4121 | Yes |
| 13 | MCM7 |  |  | 289 | 4.970 | 0.4430 | Yes |
| 14 | EZH2 |  |  | 312 | 4.888 | 0.4722 | Yes |
| 15 | RFC4 |  |  | 370 | 4.741 | 0.4979 | Yes |
| 16 | TFDP1 |  |  | 750 | 3.843 | 0.4936 | Yes |
| 17 | TMPO |  |  | 792 | 3.788 | 0.5144 | Yes |
| 18 | VRK1 |  |  | 939 | 3.570 | 0.5260 | Yes |
| 19 | DONSON |  |  | 944 | 3.561 | 0.5482 | Yes |
| 20 | CHAF1A |  |  | 971 | 3.525 | 0.5685 | Yes |
| 21 | FBXO5 |  |  | 1304 | 3.137 | 0.5633 | Yes |
| 22 | NPAT |  |  | 1318 | 3.120 | 0.5820 | Yes |
| 23 | ASF1B |  |  | 1319 | 3.119 | 0.6017 | Yes |
| 24 | PCNA |  |  | 1346 | 3.087 | 0.6193 | Yes |
| 25 | CDCA4 |  |  | 1427 | 3.018 | 0.6323 | Yes |
| 26 | RAB27A |  |  | 1438 | 3.011 | 0.6506 | Yes |
| 27 | ATAD2 |  |  | 1532 | 2.917 | 0.6620 | Yes |
| 28 | RRM2 |  |  | 2047 | 2.485 | 0.6390 | No |
| 29 | KCNK1 |  |  | 2906 | 1.983 | 0.5868 | No |
| 30 | SLBP |  |  | 2921 | 1.974 | 0.5982 | No |
| 31 | BARD1 |  |  | 3028 | 1.920 | 0.6024 | No |
| 32 | C10ORF119 |  |  | 3202 | 1.838 | 0.6010 | No |
| 33 | HUNK |  |  | 3560 | 1.680 | 0.5847 | No |
| 34 | HMGB2 |  |  | 3883 | 1.558 | 0.5703 | No |
| 35 | RMI1 |  |  | 4478 | 1.327 | 0.5339 | No |
| 36 | FANCL |  |  | 4824 | 1.213 | 0.5155 | No |
| 37 | RRM1 |  |  | 4872 | 1.197 | 0.5196 | No |
| 38 | RECQL4 |  |  | 5037 | 1.141 | 0.5144 | No |
| 39 | EED |  |  | 5267 | 1.060 | 0.5038 | No |
| 40 | E2F2 |  |  | 5382 | 1.019 | 0.5017 | No |
| 41 | SNTB2 |  |  | 5909 | 0.845 | 0.4674 | No |
| 42 | POLD3 |  |  | 6667 | 0.601 | 0.4141 | No |
| 43 | ANKRD25 |  |  | 7470 | 0.321 | 0.3557 | No |
| 44 | TMEM97 |  |  | 7575 | 0.280 | 0.3496 | No |
| 45 | FST |  |  | 8866 | -0.240 | 0.2539 | No |
| 46 | DCK |  |  | 8935 | -0.280 | 0.2506 | No |
| 47 | SUZ12 |  |  | 9542 | -0.583 | 0.2086 | No |
| 48 | PBX3 |  |  | 10267 | -1.001 | 0.1603 | No |
| 49 | CDKN1A |  |  | 10787 | -1.380 | 0.1299 | No |
| 50 | C14ORF130 |  |  | 10945 | -1.523 | 0.1277 | No |
| 51 | EGR1 |  |  | 11628 | -2.148 | 0.0899 | No |
| 52 | FAM111A |  |  | 11892 | -2.431 | 0.0854 | No |
| 53 | EPS8 |  |  | 12636 | -3.502 | 0.0516 | No |
Table: GSEA details [plain text format]

  

Fig 2: VERNELL\_PRB\_CLSTR1: Random ES distribution      
 Gene set null distribution of ES for **VERNELL\_PRB\_CLSTR1**
